# Engineering Low‐Power Cavity‐Coupled Plasmonic Nanotweezers

**DOI:** 10.1002/nap2.70318

**Published:** 2026-10-11

**Authors:** Nasrin Abdollahi, Abraham Vázquez‐Guardado

**Affiliations:** ^1^ Department of Electrical and Computer Engineering North Carolina State University Raleigh North Carolina USA; ^2^ Center for Advanced Self‐Powered Systems of Integrated Sensors and Technologies (ASSIST) North Carolina State University Raleigh North Carolina USA

**Keywords:** Brownian retention, Maxwell stress tensor, optical forces, optical potential, optical trapping, plasmonic nanotweezers

## Abstract

Plasmonic nanoapertures extend optical trapping into the subwavelength regime; but quantitative design rules remain limited because trapping is often inferred from spectral responses or field enhancement alone. This work establishes a full‐field analysis framework for designing plasmonic nanoaperture tweezers under coherent illumination and experimental restrictions. We studied the plasmonic responses of three complementary plasmonic platforms—nanohole, nanohole–nanodisk, and cavity‐coupled nanohole–nanodisk arrays—and their nanotrapping performance on dielectric and metallic nanoparticles using three‐dimensional electromagnetic simulations and Maxwell stress tensor calculations. The results show that the force landscape in the particle volume depends strongly on the plasmonic resonance strength, the nanoparticle material, and the interaction with the target nanoparticle. In particular, we found that the cavity‐coupled plasmonic array represents the most promising nanotrapping platform, capable of stable nanotrapping of metallic nanoparticles even under low power budgets and modest optical systems. Finally, we introduce a multi‐criteria figure of merit (FoM) to compare trade‐offs among trap stability, particle detuning effects, optical load, and nanoparticle material response. These results provide design guidance for compact, low‐input‐power plasmonic nanotweezer devices.

## Graphical abstract

Cavity‐coupled plasmonic nanoapertures enhance particle‐accessible near fields and enable strong local nanoparticle trapping under low input‐power conditions. Full‐field electromagnetic and Maxwell stress tensor analyses quantify particle‐dependent resonance perturbation, optical force, Brownian retention, and fixed‐wavelength force retention, providing practical design guidance for compact plasmonic nanotweezers.

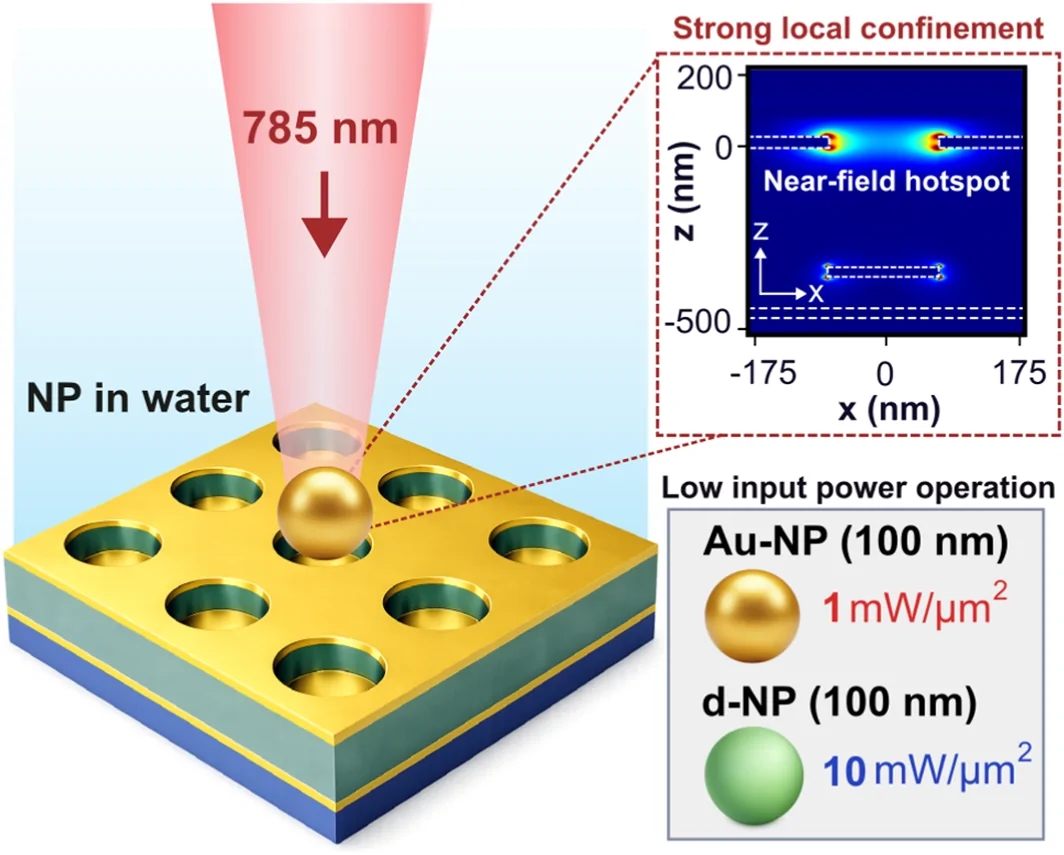

## 1 Introduction

Optical tweezers enable noncontact trapping and manipulation of microscopic objects in aqueous environments with high precision [1, 2, 3, 4]. Over the past decades, this technology has become an essential tool in science: from single‐molecule biophysics [5], cell and tissue mechanics [6], microrheology [7], soft‐matter and colloidal physics [8, 9], to quantum science with ultracold atoms and molecules [8]. However, extending conventional optical trapping to subwavelength particles in liquid environments remains a fundamental challenge in diffraction‐limited optics. As nanoparticle size decreases, their polarizability drops and the gradient force generated by diffraction‐limited optics becomes too weak to reliably overcome scattering forces and thermally driven Brownian fluctuations [10, 11]. For example, conventional far‐field optical tweezers require focal intensities on the order of 100−1000 mW/μm^2^ to trap 50 nm dielectric nanoparticles (*n* ≈ 1.59). Stable trapping becomes progressively more difficult as the nanoparticle size decreases into the deep‐subwavelength regime, motivating the development of plasmonic optical tweezers for nanoscale trapping [12, 13, 14]. In practice, this limitation often forces the use of higher optical intensities, which increases the risk of local heating leading to trap instability [1, 4, 13, 14, 15].

Plasmonic nanostructures offer a route beyond this limit. Representative examples include double‐nanohole apertures [16, 17], resonant coaxial apertures [18], metamaterials [19, 20, 21], nanohole arrays [3, 22], and hybrid plasmonic‐dielectric architectures [23]. Among them, nanohole apertures in metallic films are especially attractive because they preserve direct physical access to the trapping region above the surface [16, 17, 24, 25]. At the localized surface plasmon resonance (LSPR), these structures confine optical energy to subwavelength volumes and generate near‐field gradients stronger than those available in conventional diffraction‐limited optical tweezers [24, 26]. Across these nanoengineered systems, the geometry‐dependent near‐field distribution governs the overall trapping performance [3, 18, 19, 22, 23, 24]. In fact, plasmonic resonators like these have extended optical trapping toward deep subwavelength dimensions, including numerical predictions of trapping 5 nm dielectric nanoparticles using a plasmonic dark modeunder tightly focused radially polarized excitation (NA=1.2) and 10 nm dielectric nanoparticles using graphene plasmons at an incident intensity of ∼4.4 mW/μm^2^, as well as experimental trapping of 4 nm CdSe/ZnS quantum dots using an ultranarrow‐gap (5 nm) plasmonic nanoantenna with a 10 mW trapping beam (∼3.2 mW/μm^2^) [27, 28, 29]. Earlier, Juan et al. experimentally demonstrated self‐induced back‐action (SIBA) trapping of individual 50 nm dielectric nanoparticles using a resonant nanoaperture in a metallic film with 1.9 mW incident optical power (∼1.9 mW/μm^2^) [30]. Furthermore, hybrid electrohydrodynamic/plasmonic approaches have demonstrated trapping of ∼100 nm nanoparticles using 6.3 mW of incident power concentrated on 1.6 μm spot (3.2 mW/μm^2^) [31]. These demonstrations show that trapping deep subwavelength nanoparticles alone is not an appropriate measure of performance, because achievable trapping conditions also depend on particle material, resonant architecture, operating wavelength, and optical power requirements. Nonetheless, they provide representative practical benchmarks for array‐based nanoparticle trapping under experimental conditions.

Despite this sustained progress, quantitative design rules for plasmonic nanotweezers remain fragmented. Most studies focus on a single structure or nanoparticle class under structure‐specific conditions that make cross‐platform comparison difficult [19, 24, 32]. In addition, trapping performance is often inferred from resonance shifts, field enhancement, or simplified dipolar arguments, even though resonant scattering, particle self‐induced back‐action, and radiation pressure can reshape the full three‐dimensional force landscape [22, 30, 32, 33]. For instance, we previously reported the effects of nanoparticle material and size on the axial force and trapping potential in an aqueous environment [34, 35]. Accordingly, the contribution of the present study lies in integrating cavity‐coupled plasmonic resonance engineering, explicit particle‐loaded spectral analysis, fixed‐wavelength MST force calculations, and particle‐dependent confinement within a common full‐field design framework.

This work establishes a particle‐inclusive full‐field design framework for highly dispersive nanoaperture plasmonic systems. The adopted geometry consists of complementary plasmonic resonator arrays integrated with a photonic cavity [36, 37, 38, 39]. Parametric optimization produced a geometrical solution with an LSPR that aligns with commercial near‐infrared coherent sources (∼785 nm). We systematically analyzed uncoupled and coupled plasmonic architectures using vector‐field finite‐difference time‐domain (FDTD) simulations and time‐averaged Maxwell stress tensor (MST) analysis, to gain insights into both the optical response and nanotrapping performance. In addition, this work introduces a heuristic multi‐criteria figure of merit (FoM) as a secondary tool for comparing trade‐offs among practical operating constraints. Within this framework and FoM benchmark, the cavity‐coupled architecture provides the most favorable combination of near‐field enhancement and optical‐force response. The geometry is feasible for nanoscale prototyping and may be adaptable to nanoimprint‐based fabrication subject to process‐specific optimization, suggesting a route toward compact, low‐input‐power, on‐chip plasmonic nanotrapping platforms.

## 2 Results

### 2.1 Plasmonic Array Design and Resonance Engineering

The plasmonic platform consists of a periodic nanohole (nH) array with radius R patterned in a gold film, arranged in a square lattice with period P, supported on a dielectric substrate, and immersed in water, as shown in Figure 1a. The period was selected from an initial period–radius optimization to place the LSPR near the target 780−790 nm operating window and to provide a baseline geometry for subsequent near‐field and trapping analysis and refinement. The nH array was modified with a recessed gold nanodisk (nD) beneath the aperture, forming a nanohole–nanodisk (nHnD) array with tunable nH–nD separation $L_{d}$, as shown in Figure 1e. Finally, a gold reflector was added to form a cavity‐coupled nanohole‐nanodisk (CCnHnD) array with cavity spacing L, as shown in Figure 1i. These sequential modifications provide a parameter space for tuning the target LSPR and increasing the particle‐accessible near‐field intensity above the aperture. Rather than assuming that field enhancement alone determines trapping performance, we use the parametric analysis, summarized in Figure 1, to identify resonant operating points and then evaluate their nanotrapping relevance through accessible‐gradient and MST force analyses in the following sections. Full‐vectorial FDTD simulations with broadband, normally incident plane‐wave excitation calculated the optical response of each architecture and the exported electromagnetic fields, postprocessed in MATLAB, provided the quantitative near‐field and force analysis, as described in the Methods.

**FIGURE 1:**
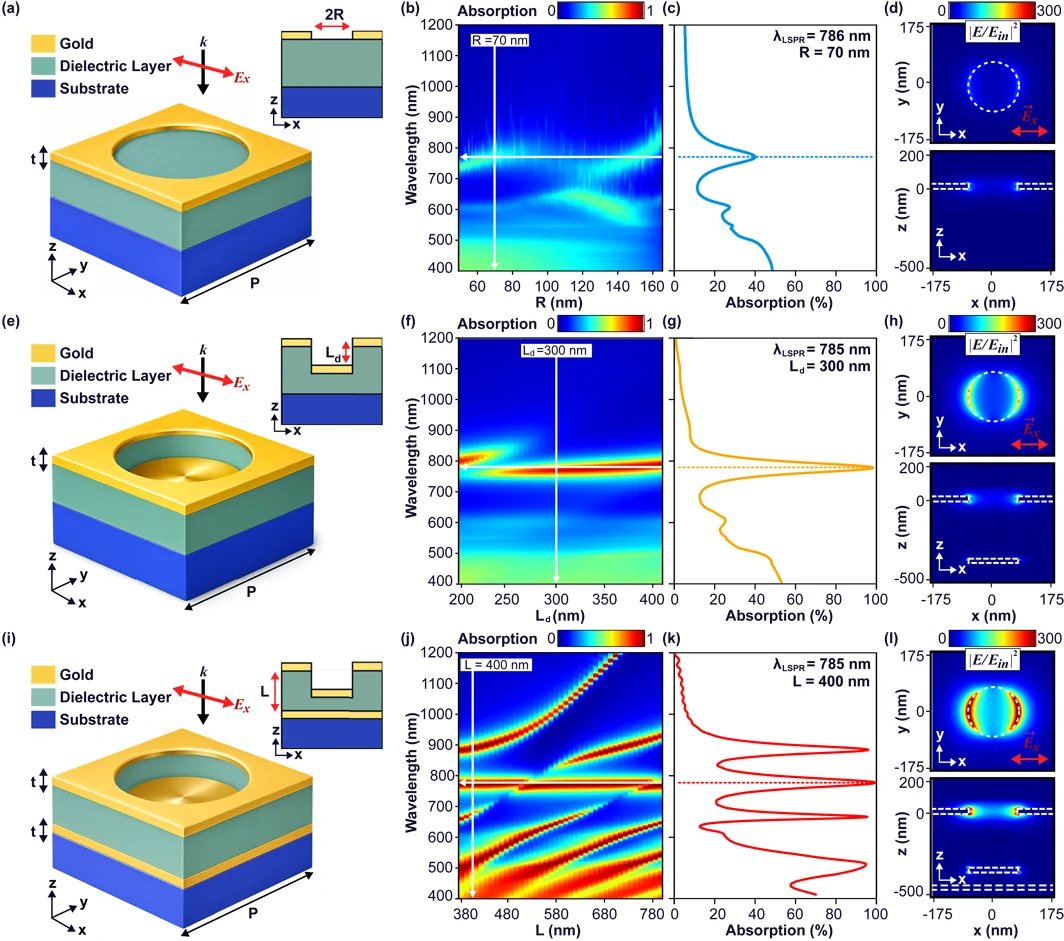
Plasmonic substrate design optimized for nanoparticle trapping. (a) Unit cell representing a nanohole (nH) square array (P=350 nm) of circular nHs with radius (R) in a gold thin film (t=30 nm) resting on a dielectric substrate (n=1.56) and immersed in an aqueous environment (n=1.33). The array is excited by a polarized plane‐wave ($E=E_{x}\hat{x}$) at a normal angle of incidence ($k=-k_{z}\hat{z}$). (b) Absorption dispersion versus nanohole radii (R=50−160 nm) showing the localized surface plasmon resonance (LSPR). (c) Absorption spectrum for an array with nH radius R=70 nm produces an LSPR around 786 nm. (d) Top‐ and cross‐sectional views of the electric near‐field intensity at the LSPR (786 nm). (e) Unit cell of an nH‐nanodisk (nHnD) array, derived from the structure in (a) in which a gold nanodisk of radius R=70 nm and thickness t=30 nm is embedded within the dielectric medium at a distance, $L_{d}$, beneath the nH. (f) Absorption dispersion versus nHnD separation ($L_{d}=200-500$ nm) showing the LSPR. (g) Absorption spectrum for a nHnD array with disk recess depth $L_{d}=300$ nm produces an LSPR around 785 nm. (h) Top‐ and cross‐sectional views of the electric near‐field intensity at the LSPR (785 nm). (i) Unit cell of a cavity‐coupled nHnD (CCnHnD) array formed by adding a second 30 nm gold layer beneath the disk. (j) Absorption dispersion versus cavity spacing (L=380−780 nm) showing a hybrid LSPR. (k) Absorption spectrum for an array with cavity spacing L=400 nm produces an LSPR around 785 nm. (l) Top‐ and cross‐sectional views of the electric near‐field intensity at the LSPR (785 nm).

The absorption dispersion of the nH array, calculated using FDTD modeling, shows that the LSPR red shifts with nH radius as shown in Figure 1b. In this periodic aperture array, the spectral position is governed by the combined effect of the effective polarizability of each nanohole and the electromagnetic coupling between neighboring holes in the lattice; increasing R modifies both the single‐hole response and the collective hole‐to‐hole interaction, thereby shifting the resonance wavelength [40, 41, 42]. Based on this dispersion, we selected R=70 nm, which places the LSPR at ∼786 nm (Figure 1c), close to a standard 785 nm near‐infrared laser line. The corresponding top‐ and cross‐sectional near‐field profiles in Figure 1d show a dipolar near‐field distribution concentrated at the aperture rim, with an intensity enhancement ${|E/E_{\text{in}}|}^{2}$ of approximately 50 at z=5 nm above the structure hotspot. This response defines the baseline near‐field performance of the nH platform: the resonance is accessible from the superstrate, but the enhancement remains moderate and is primarily localized around the aperture boundary.

The introduction of a recessed gold nD beneath the nH aperture in the nHnD array (Figure 1e) transforms the laterally coupled plasmonic mode into a hybridized vertically coupled mode. The dispersion in Figure 1f shows that the dominant control parameter is the nHnD separation ($L_{d}$). The selected operating point ($L_{d}=300$ nm) yields an LSPR near 785 nm, see Figure 1g. Together, these parameters R=70 nm and $L_{d}=300$ nm define a recessed geometry with an aspect ratio of approximately ∼2:1. Such dimensions are compatible with the nanoimprint lithography technique, where prior work has demonstrated reliable nanoimprint of nano holes (period = 560 nm, radius = 100 nm, and hole depth = 350 nm; aspect ratio of 1.75:1) over $4\times 4\;mm^{2}$ to produce plasmonic substrates for biosensing applications [43]. Furthermore, recent advancements to the nanoimprint lithography processes, including the established and commercially available roll‐to‐roll process, support the feasibility of device fabrication and deployment at scale [36, 39, 43, 44]. The near‐field profiles in Figure 1h reveal a field intensity enhancement of approximately 150 at z=5 nm. Relative to the nH array, the nHnD platform therefore strengthens local confinement up to 3‐fold and redistributes the electromagnetic energy through nH‐nD near‐field coupling.

The incorporation of the gold reflector introduces cavity modes in the CCnHnD array that hybridize with the LSPR [45, 46], as shown by the dispersion in Figure 1j. The selected cavity spacing, L=400 nm, defines an operating point with an LSPR near 785 nm (Figure 1k). The corresponding near‐field profiles in Figure 1l show the strongest field confinement among the three architectures with a peak field‐intensity enhancement of approximately 300 at z=5 nm. Compared with the nH and nHnD geometries, the CCnHnD structure concentrates the strongest near‐field intensity near the accessible aperture region. In addition, the CCnHnD configuration shows relatively weak LSPR dispersion over the explored cavity‐spacing range (Figure 1j), which helps relax fabrication tolerances.

### 2.2 Comparative Optical Response and Trapping Performance

The nH, nHnD, and CCnHnD arrays all support LSPRs within the target near‐infrared band (Figure 2a); nonetheless, resonance formation alone does not determine trapping performance. In the Rayleigh or dipole limit, the conservative gradient‐force contribution scales as $F_{\text{grad}}\propto \text{Re}\left\{\alpha \right\}\nabla E^{2}$ [2], where α is the particle polarizability and $E^{2}$ the electric field intensity. This approximation is generally reliable only for subwavelength nanoparticles, R≪λ [22, 47]; thus, it provides a physical basis for understanding how particle material and the available near‐field intensity gradient influence nanotrapping performance. Accordingly, resonances that confine optical energy within the structure are less effective for trapping than resonances that generate strong gradients in the external, particle‐accessible region.

**FIGURE 2:**
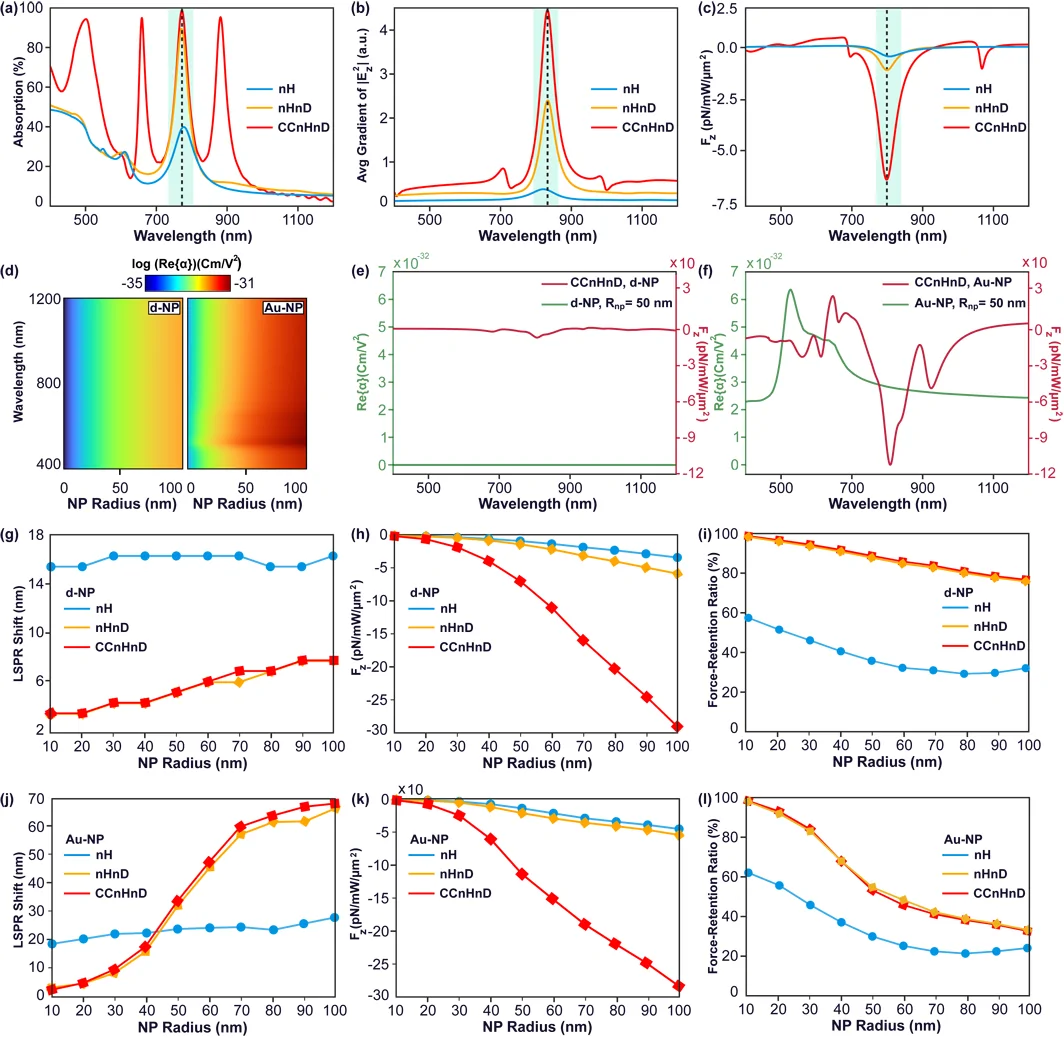
Comparative optical response and trapping performance. (a) Absorption spectra for the three plasmonic arrays (P=350 nm): nanohole (nH) array with R=70 nm, nH‐nanodisk (nHnD) array with $L_{d}=300$ nm, and cavity‐coupled nHnD (CCnHnD) with L=400 nm. (b) Volume‐averaged axial gradient $\partial E^{2}/\partial z$ evaluated in a region beginning 5 nm above the top surface of one unit cell for the three plasmonic arrays. (c) Axial force spectra $F_{z}\left(\lambda \right)$ exerted on a dielectric nanoparticle (diameter 100 nm, n=1.6) placed at the center of the unit cell for all three plasmonic arrays at (x=y=0,z=55 nm). (d) Spectra of the real part of polarizability Re{α(λ)} of dielectric (n=1.6) and gold nanoparticles for radius ($R_{\text{np}}=10-100$ nm). (e, f) Real part of polarizability Re{α(λ)} and axial force $F_{z}\left(\lambda \right)$ spectra comparison for (e) dielectric and (f) gold nanoparticles with radius $R_{\text{np}}=50$ nm for CCnHnD. (g, j) Localized surface plasmon resonance (LSPR) detuning, $\Delta \lambda_{\text{LSPR}}=\lambda_{\text{LSPR},\text{NP}}-\lambda_{\text{LSPR},0}$ for (g) dielectric and (j) gold nanoparticles placed at (x=y=0,z=55 nm). (h, k) Maximum axial attractive force $F_{z}$ at the LSPR for a (h) dielectric and (k) gold nanoparticle placed 55 nm above the top gold surface. (i, l) Effect of nanoparticle radius on the axial force at the target laser line (785 nm) with respect to the maximum attractive force at the LSPR for (i) dielectric and (l) gold nanoparticles.

Figure 2b compares the volume‐averaged axial gradient of the local intensity, $\partial E^{2}/\partial z$, evaluated from volumetric field monitors beginning 5 nm above the top gold surface, as described in the Methods. Consistent with this picture, the peak volume‐average axial gradient reaches approximately 0.5, 2.2, and 4.5 for the nH, nHnD, and CCnHnD arrays, respectively. Furthermore, strong resonances supported in the CCnHnD (∼670 and ∼900 nm), corresponding to cavity‐coupled hybrid LSPR modes buried within the cavity geometry (Supporting Information S1: Figure S1), do not produce near‐fields relevant for nanotrapping (Figure 2c).

The axial force spectrum, exerted on a 100 nm dielectric nanosphere (n=1.6) positioned at the center of the unit cell, further supports this performance ranking (Figure 2c). The peak attractive axial force increases from approximately −0.5 pN/(mW/μm^2^) for the nH array to about −1.3 pN/(mW/μm^2^) for the nHnD array and to about −6.3 pN/(mW/μm^2^) for the CCnHnD array. These values correspond to an enhancement of roughly 3× from nH to nHnD, 13× from nH to CCnHnD, and 5× from nHnD to CCnHnD. This comparison confirms that spectral response alone is not a sufficient design criterion: a structure may appear favorable because of strong absorption while still trapping poorly if the optical energy is concentrated deep within the geometry. In contrast, the CCnHnD architecture provides the strongest surface‐accessible field gradient and the largest usable axial force near target experimental lasers (785 nm). This enhanced response is consistent with previous demonstrations of cavity‐enhanced plasmonic devices [45, 46, 48, 49].

### 2.3 Particle‐Dependent Spectral Response and Force Retention

The trapping response of the selected CCnHnD array depends on nanoparticle polarizability and size. Figure 2d highlights the real part of the polarizability, Re{α}, because in the Rayleigh limit, it governs the conservative gradient‐force contribution, whereas Im{α} is associated with the scattering and absorption components of the optical force [50, 51]. This polarizability‐based argument is used here only to interpret the material trend; the optical forces reported below are calculated directly from the full MST analysis. At 785 nm, Re{α} for the 100 nm dielectric nanoparticle (d‐NP) is on the order of $10^{-33}$, whereas that of the 100 nm gold nanoparticle (Au‐NP) is on the order of $10^{-32}$; approximately one order of magnitude larger than the d‐NP. Importantly, the isolated spherical gold nanoparticle has its natural dipolar plasmon resonance in the visible spectral region, around 520‐550 nm depending on nanoparticle size and surrounding medium, rather than near the 785 nm operating wavelength of the CCnHnD array [52]. Therefore, the enhanced force obtained for the Au‐NP should not be interpreted as direct resonant coupling between the isolated nanoparticle plasmon and the plasmonic array mode. Instead, it reflects the stronger polarizability and stronger electrodynamic perturbation of the cavity‐supported near‐field response by the metallic nanoparticle. This distinction is evident in Figure 2e,f: for representative 100 nm nanoparticles, the maximum attractive axial force reaches approximately −7 pN/(mW/μm^2^) for the d‐NP at the shifted resonance, whereas it increases to approximately −100 pN/(mW/μm^2^) for the Au‐NP. Thus, although the real component of the polarizability differs by about one order of magnitude at 785 nm, the full‐field MST calculation predicts a larger force contrast because the metallic nanoparticle modifies and samples the resonant near‐field of the CCnHnD array.

The nanoparticle size dispersion in Figure 2g–i for d‐NPs and Figure 2j–l for Au‐NPs, radii ranging from 10 to 100 nm, resolve three experimental observables: (1) the LSPR shift relative to the nanoparticle‐free structure, (2) the maximum attractive axial force at the particle‐detuned resonance, and (3) the force‐retention ratio at the operating laser wavelength (785 nm). Here, the force‐retention ratio, $R_{F}$, is defined as the ratio between the attractive axial optical force retained at the operating laser wavelength $F_{z}\left(785\;\text{nm}\right)$ and the maximum attractive axial force expected at the particle‐detuned LSPR $F_{z,\max}$, $R_{F}=|F_{z}\left(785\;\text{nm}\right)|/|F_{z,\max}|$. Therefore, $R_{F}$ quantifies how much of the trapping force remains experimentally available when the system is operated with a fixed‐wavelength laser rather than tuned to the particle‐shifted resonance. A value close to unity indicates that the particle‐loaded resonance still provides nearly maximum axial attraction at the laser line.

For d‐NPs, the LSPR response evolves smoothly with nanoparticle radius producing a spectral shift from 2 to 6 nm in both nHnD and CCnHnD arrays and a nearly flat dispersion in the nH array (Figure 2g), whereas the maximum attractive axial force in the CCnHnD structure is about 10‐fold larger than in the nH structure (Figure 2h). Au‐NPs exhibit a markedly stronger and more dispersive response, with the LSPR shift increasing from 3 to 67 nm in both nHnD and CCnHnD arrays and from 18 to 28 nm in the nH array (Figure 2j), corresponding to an approximately 9‐fold larger detuning and a 15‐fold stronger peak attraction (Figure 2k) than in the dielectric case over the same nanoparticle size range. This contrast is consistent with the larger and more dispersive polarizability of gold. The same distinction appears in the fixed‐wavelength response: for a representative 100 nm d‐NP, the force‐retention ratio at 785 nm remains about 85% (Figure 2i), whereas for a 100 nm Au‐NP, it is about 60% (Figure 2l), indicating a stronger sensitivity of gold to particle‐induced detuning because the optimum‐force wavelength is red‐shifted further from the laser line. Taken together, these trends show that detuning alone is not the most appropriate experimental metric; rather, the practically relevant quantity is force retention under fixed‐wavelength operation. In this sense, the CCnHnD architecture remains the most promising of the three platforms because it combines the strongest axial attraction with the most favorable balance between detuning and usable force at the operating wavelength for both nanoparticle materials. The remainder of this manuscript therefore focuses on the CCnHnD geometry.

### 2.4 Spatial LSPR Detuning and Axial Force

A nanoparticle approaching the nanoaperture of the CCnHnD array detunes the LSPR in proportion to its location and material. This spatially and material‐dependent detuning shifts the wavelength at which the system sustains the maximum axial force. Therefore, when using a narrowband laser (785 nm ± 1 nm) as the excitation source, the particle‐loaded CCnHnD system operates under suboptimal conditions (see Methods for more details). This behavior is consistent with particle‐induced back‐action, in which the nanoparticle perturbs the cavity‐supported resonance and the resulting resonance shift feeds back into the optical force experienced by the particle [3, 30, 53, 54]. The present static analysis does not establish a new back‐action dynamical trapping regime, but rather it quantifies how particle‐induced resonance perturbation affects fixed‐wavelength optical‐force performance.

The d‐NP displays a modest resonance perturbation, which is attributed to its weaker and less dispersive polarizability. As shown in Figure 3a,b, the LSPR spatial detuning and axial force are largest near the aperture opening and decrease as the nanoparticle moves away from the surface, consistent with the evanescent near‐field produced in plasmonic resonators. Here, z denotes the location of the 100 nm nanoparticle center at the center of the unit cell and above the top gold surface. At this closest separation, the d‐NP shifts the LSPR by only ∼15 nm, up to ∼16 nm when positioned at the hot spots; nonetheless, the axial force is −5.3 pN/(mW/μm^2^), and −15 pN/(mW/μm^2^) at the hot spots. In contrast, the Au‐NP produces stronger material‐ and position‐dependent perturbation. As shown in Figure 3d,e, the maximum detuning reaches ∼45 nm at the closest nanoparticle‐edge clearance and center of the unit cell, about 3‐fold larger than the d‐NP response under the same operating condition. This detuning increases to ∼70 nm at the hot spots. The corresponding axial force is −68 and −100 pN/(mW/μm^2^) at the hot spot, which is approximately 13‐fold and 7‐fold larger than that of the d‐NP, at both comparative locations. This larger detuning indicates strong electrodynamic coupling between the metallic nanoparticle and the cavity‐supported near‐field, making the Au‐NP force response more sensitive. The tomographic maps in Figure 3c–f show the nanoparticle‐loaded spatial axial attractive force $F_{z}$ exerted on the nanoparticle at the operating laser wavelength (785 nm), not at the particle‐shifted LSPR. In both cases, the strongest attractive force is located near the aperture opening and close to the hot spots, where the accessible near‐field gradient is the strongest. To further examine the spatial range of the optical interaction, the axial FDTD/MST force calculations in Figure 3b,e were extended to nanoparticle centered up to z=500 nm above the top gold surface. For both nanoparticle materials, the attractive axial force decreases exponentially as the nanoparticle moves away from the aperture, confirming that the strong optical interaction provided by the CCnHnD architecture is spatially localized near the plasmonic surface. Accordingly, the force, potential, and stiffness analyses presented here characterize local confinement after the nanoparticle has reached the plasmonic near‐field region (∼150 nm particle center to top gold surface), which evidences the inherent limitation in plasmonic‐based nanotrapping platforms.

**FIGURE 3:**
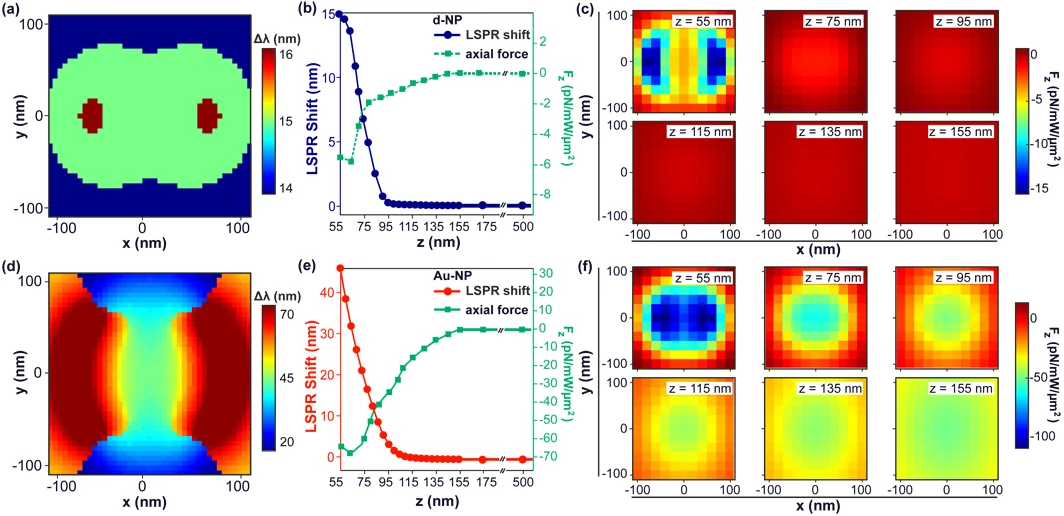
Trapping effects on LSPR detuning and axial force. (a–c) Dielectric nanoparticle (d‐NP, $R_{\text{np}}=50$ nm): (a) Localized surface plasmon resonance (LSPR) transversal detuning caused by the d‐NP with its center located at 55 nm above the CCnHnD array, (b) LSPR longitudinal detuning and axial trapping force $F_{z}\left(z\right)$ caused by the d‐NP located at the center of the unit cell (x=y=0 nm), and (c) tomographic maps of the axial trapping force $F_{z}\left(x,y\right)$ exerted on d‐NP placed at different z planes. (d–f) Gold nanoparticle (Au‐NP, $R_{\text{np}}=50$ nm): (d) LSPR transversal detuning (x,y) caused by the Au‐NP located at (x=y=0,z=55 nm) above the CCnHnD array, (e) LSPR longitudinal detuning and axial trapping force $F_{z}\left(z\right)$ caused by the Au‐NP located at the center of the unit cell (x=y=0 nm), and (f) tomographic maps of the axial trapping force $F_{z}\left(x,y\right)$ exerted on Au‐NP placed at different z planes.

### 2.5 Trapping Characteristics at the Operating Laser

The force and potential landscapes at the target operating wavelength provide complementary measures of local optical confinement. Figure 4a shows the geometry used to compute the spatially dependent optical forces for 100 nm dielectric and metallic nanoparticles. The optical forces and potential were calculated at the fixed excitation wavelength of 785 nm using MST analysis, as described in the Methods.

**FIGURE 4:**
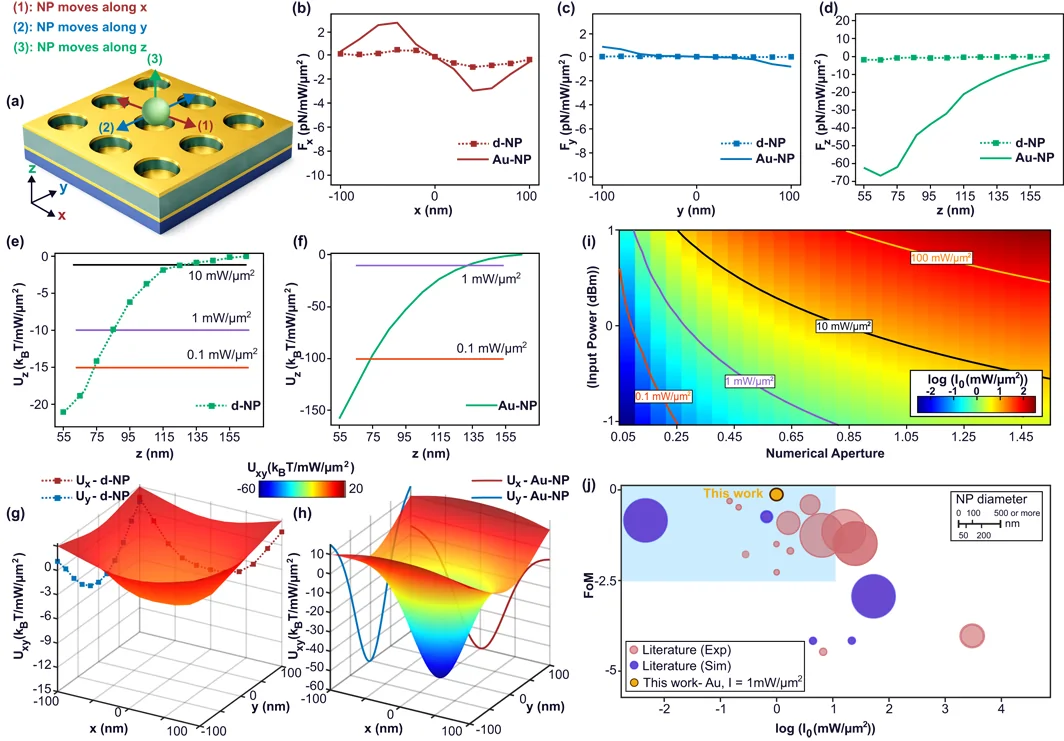
Trapping characteristics of the CCnHnD array. (a) Graphic representation of nanoparticle ($R_{\text{np}}=50$ nm) spatial positioning above the CCnHnD array showing the axial directions used in the potential calculations. (b, c) Lateral forces $F_{x}\left(x,y=0\right)$ and $F_{y}\left(x=0,y\right)$ exerted on gold (Au‐NP) and dielectric (d‐NP) located at z=55 nm. (d) Axial force $F_{z}\left(z\right)$ exerted on Au‐NP and d‐NP located at the center of the unit cell (x=y=0 nm). (e, f) Axial potential $U_{z}\left(z\right)$ of the d‐NP and Au‐NP located at the center of the unit cell. (g, h) Reconstructed transverse potential U(x,y) for the d‐NP and Au‐NP located at z=55 nm. (i) Focal intensity $I_{0}$ of a Gaussian beam generated using a focusing system with numerical aperture (NA) and a laser (785 nm) at different input powers. (j) Figure of merit, $\text{FoM}=\log_{10}\left(\text{SHRP}\right)$, plotted versus optical intensity for representative plasmonic nanotrapping studies. Marker color indicates study type (experiment vs. simulation), whereas size scales with nanoparticle diameter. The highlighted marker denotes this work for the 100 nm Au‐NP + CCnHnD case. The shaded region serves as a visual guide to cases combining relatively low optical intensity with larger FoM values under the present heuristic definition.

The lateral forces in Figure 4b,c and the axial force in Figure 4d show a clear effect of the nanoparticle's material on the nanotrapping performance. The differences in force translate directly into the optical potentials in Figure 4e,f, where the d‐NP exhibits a shallower and weaker curved landscape, whereas the Au‐NP produces deeper and more anisotropic confinement around the center of the CCnHnD. The corresponding transverse potential minima are approximately −4 *k*_*B*_ *T*/(mW/μm^2^) for the d‐NP and −60 *k*_*B*_ *T*/(mW/μm^2^) for the Au‐NP (Figure 4g,h). The axial potential minima are likewise approximately −20 *k*_*B*_ *T*/(mW/μm^2^) for the d‐NP and −160 *k*_*B*_ *T*/(mW/μm^2^) for the Au‐NP (Figure 4e,f). This contrast becomes quantitative when the force and potential are normalized per unit of optical intensity (mW/μm^2^), providing a direct reference frame for experimental design. At the reference irradiance of 1 mW/μm^2^, the Au‐NP satisfies the axial Brownian‐retention criterion and exhibits lateral stiffnesses of approximately $k_{x}=72$ and $k_{y}=67\;\text{fN}/\text{nm}$. The root‐mean‐square (RMS) thermal localization widths, $\sigma_{i}$ where *i* represents the transverse coordinate as described in the Methods, were used to quantify the spatial extent of local confinement. This analysis estimates near‐equilibrium thermal localization of $\sigma_{x}\approx 7.6$ nm and $\sigma_{y}\approx 7.9$ nm. Under the same reference irradiance, the d‐NP exhibits substantially weaker stiffness, $k_{x}\approx 14.8$ and $k_{y}\approx 7.4\;\text{fN}/\text{nm}$, corresponding to broader thermal localization widths of approximately $\sigma_{x}\approx 16.7$ and $\sigma_{y}\approx 23.7$ nm, respectively, while remaining below the axial Brownian‐retention criterion. At $10\;\text{mW}/\mu m^{2}$, the d‐NP satisfies the axial retention condition, and its thermal localization widths decrease to approximately $\sigma_{x}\approx 5.3$ and $\sigma_{y}\approx 7.5$ nm. Thus, at the common 1 mW/μm^2^ reference irradiance, the Au‐NP exhibits substantially tighter near‐equilibrium lateral confinement than the d‐NP [10, 11, 55]. Specifically, at the same intensity, the Au‐NP provides approximately 5‐fold larger stiffness along the polarization direction and 9‐fold larger stiffness along the orthogonal direction compared with the d‐NP. When the d‐NP irradiance is increased to 10 mW/μm^2^, both particle cases exhibit nanoscale lateral thermal localization at their respective Brownian‐retention conditions, whereas the Au‐NP reaches the retention condition at an irradiance 10 times smaller. Although the absolute stiffness remains governed by the polarization‐dependent near‐field profile, the larger (Au‐NP/d‐NP) enhancement in y indicates that the material response strengthens confinement in both transverse directions. These results show that the material dependence is not limited to a larger attractive force; it determines whether the trap can simultaneously provide axial retention and lateral confinement within a realistic optical‐intensity range. The 1 mW/μm^2^ condition is used here as a reference for comparing the nanoparticle materials and intensity‐normalized nanotweezer benchmarks rather than a definition of the minimum Au‐NP trapping irradiance. Within the linear electromagnetic model considered here, the optical force, potential‐energy scale, and stiffness scale linearly with incident irradiance [1, 56]. Consequently, lower irradiances produce weaker confinement, with the thermal localization width scaling approximately as $\sigma_{i}\propto I^{-1/2}$. As the irradiance is reduced, the optical force, potential depth, and stiffness decrease proportionally. Local confinement is retained only while the corresponding axial potential barrier remains above the adopted Brownian‐retention threshold.

For experimental implementation, nanoparticles outside the localized near‐field region may reach the trapping site through Brownian diffusion or auxiliary transport mechanisms [24]. Previous plasmonic‐tweezer studies have addressed this limited interaction range using electrohydrodynamic, electrothermoplasmonic, or dielectrophoretic transport to deliver nanoparticles toward the plasmonic trapping region [31, 57, 58]. These approaches provide established routes for coupling longer‐range particle delivery with the local optical confinement regime considered here.

A practical prospective operating regime is summarized in Figure 4i, which relates the input optical power of a 785 nm laser and the numerical aperture (NA) of the focusing system to the diffraction‐limited focal irradiance, using the Gaussian‐beam approximation described in the Methods section. Here, “low‐power” refers specifically to the required input laser power rather than to a low local irradiance at the trapping plane. Under the adopted focusing model, the Au‐NP reaches the axial Brownian‐retention condition at a focal irradiance of approximately 1 mW/μm^2^, corresponding to an input power of approximately 1 mW at NA=0.25. In contrast, the d‐NP requires a substantially higher focal irradiance of approximately 10 mW/μm^2^, corresponding to approximately 10 mW at the same NA, to satisfy the same axial‐retention criterion. Thus, the Au‐NP case reduces the required input‐power budget for achieving local optical confinement, whereas the d‐NP requires either greater input power, a higher‐NA focusing system, or a combination of both to reach the necessary focal irradiance. Importantly, these conditions characterize local optical confinement once a nanoparticle reaches the plasmonic near‐field region and do not establish deterministic long‐range loading or capture from bulk solution.

The Brownian‐retention analysis presented here is evaluated under an isothermal assumption and therefore describes optical confinement rather than the complete nonisothermal trapping dynamics expected experimentally. Optical absorption in metallic nanostructures can generate localized heating and temperature gradients even at moderate incident irradiances, especially at the LSPR [59]. Such temperature changes may modify the local thermal energy, fluid viscosity, Brownian diffusion, and introduce thermophoretic forces that can either assist or oppose optical confinement [60].

### 2.6 Performance Benchmarking and Design Implications

Here, we introduce a figure of merit (FoM) metric to serve as a performance benchmark relevant to fixed‐wavelength nanoparticle trapping. The FoM is not a fundamental physical observable parameter, and it does not intend to replace the comprehensive nanotrapping performance analysis for new designs. The FoM combines four dimensionless factors: trap‐stability factor (S), force retention ratio (R), intensity‐based optical‐load factor (H), and nanoparticle material response (P). This metric is defined in Equation (15); the individual factors are described in the Methods section. Although S, H, and R range from 0 to 1, the response factor P for metallic particles considered here ranges from 0 to 1.52. Within this definition, FoM approaching 0.18 indicates the most favorable balance of factors that predict excellent performance. Because this metric depends on the availability of benchmark data, the estimated FoM values are interpreted comparatively rather than as an absolute ranking of performance. The figure of merit for the 100 nm Au‐NP + CCnHnD model is FoM=−0.63. This score is attributed to the cavity‐enhanced trapping performance achieved at low optical intensity (1 mW/μm^2^). The axial trap‐stability factor is S=0.499, which indicates that the optical potential is sufficient to satisfy the Brownian retention criterion. The intensity‐based optical‐load factor H reflects the optical irradiance required for the selected operating condition, with larger H corresponding to a lower optical‐load requirement. Importantly, H is used only as an irradiance‐based comparative penalty and should not be interpreted as a calculation of local temperature rise or thermal safety. The force retention is R=0.6, which indicates 60% of the axial force expected at the particle‐detuned plasmonic array is available at the operating laser wavelength. Finally, the metallic nanoparticle material response is P=1.234. For comparison under the same conditions, the figure of merit for the 100 nm d‐NP + CCnHnD model is FoM≈−2.31(S≈0.083,H=0.630,R=0.85,P=0.125) when operating at I=1 mW/μm^2^. Under this condition, the d‐NP has a comparable intensity‐based optical‐load factor. However, it does not satisfy the Brownian‐retention criterion because of its weaker trap stability and smaller material response. The higher‐intensity d‐NP case, I=10 mW/μm^2^, is therefore used only as a stability‐threshold reference, where the Brownian‐retention condition is recovered (S=0.495) but with a stronger optical‐load penalty (H=0.14), giving FoM≈−2.12.

The FoM introduced here provides a comparison metric against plasmonic nanotrapping platforms reported in the literature. For consistency, benchmark inputs were taken directly from reported values whenever available. When trap depth was not reported but stiffness was available, the trap‐stability factor was calculated using the harmonic approximation described in Methods. If a required value was unavailable, we used the baseline assumptions listed in Supporting Information S1: Table S1 only to complete the FoM calculation. These cases should therefore be interpreted as good‐faith estimated benchmark entries rather than fully reported experimental or simulation values. We compare the present d‐NP and Au‐NP results with the representative experimental and simulation‐based benchmarks summarized in Supporting Information S1: Table S1. For example, the bowtie‐aperture fiber‐tip nanotweezer in Ref. [61] considers a smaller 30 nm quantum dot at 15.6 mW/μm^2^ with an estimated effective stiffness of 0.245fN/nm, FoM≈(−2.56). In comparison, the present 100 nm Au‐NP + CCnHnD operates at approximately one order of magnitude lower intensity while providing more than two orders of magnitude higher transverse stiffness. The closed–packed gold nanodisk array in Ref. [62] reports a stiffness of 727fN/nm for larger 250/350 nm polystyrene nanoparticles at 83 mW/μm^2^, FoM≈(−2.90). Resonant metamaterials provide additional low‐intensity benchmarks. Kotsifaki et al. [19] demonstrated Fano‐resonance‐assisted trapping of 20 nm dielectric nanoparticles at 930 nm and 0.23 mW/μm^2^, with an experimentally measured trap stiffness of 1.99fN/nm and an estimated FoM≈−1.44. Bouloumis et al. [3] subsequently demonstrated SIBA‐assisted trapping of 20 nm Au nanoparticles in a Fano‐resonant metamaterial at 930 nm and 0.61 mW/μm^2^; the selected operating condition corresponds to an effective stiffness of approximately 1.78fN/nm and an estimated FoM≈−0.59. In contrast, our d‐NP case remains as an important internal comparison, despite experiencing weaker optical coupling and requiring higher intensity to achieve the same axial‐retention condition. Therefore, the highlighted “This work” marker in Figure 4j corresponds to the 100 nm Au‐NP + CCnHnD case, which satisfies the low‐optical‐budget retention condition under the modeled parameters. The blue shaded region in Figure 4j represents a target operating domain combining relatively low incident irradiance with comparatively favorable FoM values under the present metric.

This comparison shows that the advantage of the CCnHnD design is not simply stronger field enhancement or stronger absorption. Rather, the cavity‐coupled aperture mode preserves a usable force‐and‐stability landscape in the particle‐accessible region at the target laser wavelength. The FoM therefore serves only as a fair practical benchmark for fixed‐wavelength nanotweezers by penalizing trade‐offs that can be missed when a platform is judged by a single metric alone, including shallow potential depth, reduced force retention at the laser wavelength, high optical intensity and unfavorable particle‐dependent optical response. It should not be interpreted as a universal ranking of nanotweezer performance.

## 3 Conclusion

This work establishes a full‐field framework to study and engineer plasmonic nanotweezer platforms. The central result is that trapping performance is governed not only by the optical response of the plasmonic array but also by particle‐dependent and practical operating requirements. Among the three architectures investigated, the CCnHnD configuration provides the most favorable combination of particle‐accessible near‐field enhancement, optical force, and fixed‐wavelength force retention, while retaining a strong response in the presence of nanoparticle‐induced resonance perturbation. Nanoparticle material also plays an important role in determining the confinement conditions. For the 100 nm Au‐NP, the local Brownian‐retention criterion is satisfied at approximately 1 mW/μm^2^, corresponding, under the adopted focusing model, to an input power of approximately 1 mW with a 0.25 NA optical system, whereas the d‐NP requires a substantially higher irradiance to reach the same retention condition. The introduced FoM provides a secondary heuristic comparison of trade‐offs among trap stability, fixed‐wavelength force retention, optical load, and nanoparticle material response, whereas the direct force, potential, and stiffness calculations remain the primary physical measures of trapping performance. More broadly, these results provide design guidance for low‐input‐power, on‐chip plasmonic nanotweezers for nanoparticle manipulation, sorting, sensing, and future active or tunable plasmonic platforms without relying solely on increased optical power.

## 4 Methods

### 4.1 Full‐Wave Electromagnetic Simulations

All electromagnetic simulations were performed using a three‐dimensional finite‐difference time‐domain (FDTD) solver in Lumerical FDTD 2024 R1.2 (Ansys Inc.). The simulated structures consisted of periodic nanohole (nH), nanohole–nanodisk (nHnD), and cavity‐coupled nHnD (CCnHnD) arrays defined on a square lattice with period P=350 nm. The top gold film thickness was 30 nm. For the selected operating geometries, the nanohole radius was R=70 nm, the nanohole–nanodisk separation was $L_{d}=300$ nm, and the cavity spacing in the CCnHnD structure was L=400 nm. Dielectric nanoparticles were modeled as polystyrene (n≈1.59), and metallic nanoparticles were modeled as gold. Gold was modeled using the Johnson and Christy optical constants [63], the substrate was defined as glass (n=1.56), and the surrounding medium was water (n=1.33).

Periodic boundary conditions were applied along the x‐ and y‐directions on a unit cell, and perfectly matched layers were used along the z‐direction. The structures were excited from the water side at normal incidence by an x‐polarized plane‐wave over a wavelength range of 400–1200 nm. A global mesh size of 5 nm × 5 nm × 5 nm was used, with a refined mesh of 2 nm × 2 nm × 2 nm in the nanoparticle and near‐surface regions. Transmission and reflection monitors were used to calculate the absorption spectrum as A=1−R−T. Field monitors in the xy, xz, and yz planes were used to export the complex electric and magnetic field vectors. For force calculations, each nanoparticle was surrounded by six field monitors defining a closed cubical surface for MST integration.

Nanoparticles were introduced individually as spherical dielectric or gold nanoparticles. Unless otherwise stated, the trapping calculations used a nanoparticle radius of $R_{\text{np}}=50$ nm, corresponding to a 100 nm diameter nanoparticle. For size‐dependent calculations, the nanoparticle radius was swept over the specified range.

### 4.2 Near‐Field Metrics

The local electric‐field intensity was calculated from the complex field components such as:

(1) $$E^{2}\equiv {\left|E\left(x,y,z\right)\right|}^{2}={\left|E_{x}\left(x,y,z\right)\right|}^{2}+{\left|E_{y}\left(x,y,z\right)\right|}^{2}+{\left|E_{z}\left(x,y,z\right)\right|}^{2}.$$

When normalized field maps were required, this quantity was divided by ${\left|E_{\text{in}}\right|}^{2}$. The near‐field quantities used for geometry comparison were extracted from a three‐dimensional monitoring volume centered on the unit cell. For the particle‐accessible region, the volume was evaluated beginning at the first clearance plane above the top gold surface. The integrated field intensity descriptor was obtained by summing $E^{2}$ over the selected volume, and the axial near‐field‐gradient descriptor was calculated as the volume‐averaged value of $\partial E^{2}/\partial z$. The axial gradient was approximated numerically as:

(2) $$\frac{dE^{2}}{dz}\approx \left(E^{2}\left(z+\Delta z\right)-E^{2}\left(z\right)\right)/\Delta z,$$

where Δ*z* is the grid spacing along the *z*‐axis.

### 4.3 Particle Polarizability

The wavelength‐dependent nanoparticle polarizability was calculated for spherical nanoparticles of radius a embedded in water. In the Rayleigh limit, the induced dipole moment is [2, 64]

(3) $$p\left(\lambda \right)=\alpha \left(\lambda \right)\;E_{\text{loc}}\left(\lambda \right),$$

where α(λ) is the nanoparticle polarizability and $E_{\text{loc}}$ is the local electric field. The quasistatic Clausius–Mossotti polarizability ($\alpha \equiv \alpha_{\text{CM}}$) was calculated as [65]

(4) $$\alpha_{\text{CM}}\left(\lambda \right)=4\pi {\varepsilon_{0}\varepsilon_{m}a}^{3}\;\frac{\varepsilon_{p}\left(\lambda \right)-\varepsilon_{m}}{\varepsilon_{p}\left(\lambda \right)+2\varepsilon_{m}},$$

where $\varepsilon_{0}$ is the permittivity of free space, $\varepsilon_{p}\left(\lambda \right)$ is the wavelength‐dependent nanoparticle permittivity of the nanoparticle and $\varepsilon_{m}$ is the relative permittivity of the surrounding medium. The real and imaginary parts of $\alpha_{\text{CM}}$ were extracted for dielectric and gold nanoparticles over the wavelength range used in the FDTD simulations. These polarizability values were used only as particle‐response descriptors; all reported optical forces were calculated from the MST.

### 4.4 Maxwell Stress Tensor Force Calculations

All trapping forces were evaluated directly from the time‐averaged MST. For a linear, isotropic medium with permittivity $\varepsilon_{m}$ and permeability $\mu_{m}$, the MST components $\langle T_{ij}\rangle$ are defined by [66]:

(5) $$\langle T_{ij}\rangle =\frac{1}{2}\text{Re}\left[\varepsilon_{0}\varepsilon_{m}\left(E_{i}E_{j}^{*}-\frac{1}{2}\delta_{ij}|E{|}^{2}\right)+\mu_{0}\mu_{m}\left(H_{i}H_{j}^{*}-\frac{1}{2}\delta_{ij}|H{|}^{2}\right)\right],$$

with i,j∈{x,y,z}, $\delta_{ij}$ the Kronecker delta, ${\left|E\right|}^{2}=E_{x}^{2}+E_{y}^{2}+E_{z}^{2}$ and ${\left|H\right|}^{2}=H_{x}^{2}+H_{y}^{2}+H_{z}^{2}$. The total force was obtained from the closed‐surface integral: $\langle F\rangle =\oint_{S}\langle \overset{⃡}{T}\rangle \cdot \hat{n}\;dA$, where $\hat{n}$ is the outward normal vector and dA is the surface element. The six enclosing field monitors were exported from Lumerical FDTD and postprocessed in MATLAB (The MathWorks Inc.) to obtain the three Cartesian force components $F_{x}$, $F_{y}$, and $F_{z}$.

### 4.5 Resonance Detuning and Fixed‐Wavelength Force Spectra

Each combination of nanoparticle material, size, and location required one FDTD simulation to determine the new particle‐detuned LSPR and the nanotrapping response. The LSPR shift was calculated as:

(6) $$\Delta \lambda_{\text{LSPR}}=\lambda_{\text{LSPR},\text{NP}}-\lambda_{\text{LSPR},0},$$

where $\lambda_{\text{LSPR},\text{NP}}$ is the LSPR wavelength with the nanoparticle present and $\lambda_{\text{LSPR},0}$ is the LSPR wavelength of the nanoparticle‐free structure. Axial force spectra were extracted from the same FDTD simulation results using MST analysis. For each case, the ratio between the axial force retained at the operating laser wavelength λ=785 nm to the maximum attractive axial force produced the spatial force maps.

### 4.6 Optical Potential Reconstruction

Numerical line integration of the force fields calculated the optical potential energy. For a nanoparticle moving from reference position $r_{0}$ to position r, the potential is ref. [2]:

(7) $$U_{r}\left(r\right)=-\int_{r_{0}}^{r}F\left(r^{\prime }\right)\cdot dr^{\prime }.$$

Axial potentials were reconstructed from $F_{z}\left(z\right)$ line integration along the z‐direction at the center of the unit cell (x=y=0) and transverse potentials were reconstructed from the two‐dimensional lateral force field at a fixed nanoparticle height $\left(z_{0}\right),$ ($F_{x}\left(x,y,z_{0}\right)$ and $F_{y}\left(x,y,z_{0}\right)$).

### 4.7 Stiffness and Brownian‐Retention Analysis and Localization

Trap stiffness was obtained by linearizing the force near the equilibrium position $r_{0}$ [1, 56, 67]:

(8) $$k_{r}=-{\frac{dF_{r}}{dr}|}_{r_{0}}.$$

The lateral stiffness values were calculated as:

(9) $$k_{x}=-{\frac{\partial F_{x}}{\partial x}|}_{x_{0}},k_{y}=-{\frac{\partial F_{y}}{\partial y}|}_{y_{0}}.$$

The effective lateral stiffness was then defined as

(10) $$k_{xy}=\sqrt{k_{x}k_{y}}.$$

This geometric‐mean definition provides a single representative stiffness for transverse localization while still requiring confinement in both lateral directions.

Axial Brownian‐retention was assessed from the axial trap depth, defined as the optical potential‐energy increase required to move the nanoparticle from the near‐surface trapped position to a selected axial escape position [53, 67]:

(11) $$\Delta U_{z}=U\left(z_{\text{escape}}\right)-U\left(z_{\text{near}}\right),$$

where $z_{\text{near}}$ was the closest simulated particle‐edge clearance above the surface and $z_{\text{escape}}$ was the selected axial escape distance. Brownian‐retention was assessed using the criterion [67]:

(12) $$\Delta U_{z}≳10k_{B}T.$$

In the present analysis, T is constant, 300 K, across space; therefore, the Brownian‐retention criterion represents an isothermal assessment of optical confinement rather than a prediction of trapping stability under nonisothermal experimental conditions. The same uniform temperature is used for the thermal localization estimates in Equation (13).

The stiffness values and axial energy barriers were normalized against the optical intensity when intensity‐normalized comparisons were required. All reported force and potential values were normalized to the incident optical intensity at the nanoparticle plane unless otherwise stated.

The spatial extent of near‐equilibrium lateral confinement was additionally estimated from the stiffness using the local harmonic approximation:

(13) $$\sigma_{i}=\sqrt{\frac{k_{B}T}{k_{i}}},i=x,y,$$

where $\sigma_{i}$ is the RMS thermal localization width about the equilibrium position. This quantity describes the expected thermal positional fluctuations within the locally harmonic optical potential and does not refer to the spatial profile of the incident optical field. For the linear electromagnetic model used here, $k_{i}\propto I$, and therefore $\sigma_{i}\propto I^{-1/2}$. The periodic full‐wave calculations use plane‐wave excitation, whereas the Gaussian focusing model, introduced separately below, is used only to relate experimental input laser power and numerical aperture to focal irradiance.

### 4.8 Focal‐Plane Irradiance Calculation

We calculated the focal‐plane irradiance $I_{0}$ using a diffraction‐limited Gaussian beam approximation,

(14) $$I_{0}=\frac{2\pi \eta P_{\text{in}}n_{\text{water}}^{2}{\text{NA}}^{2}}{\lambda_{0}^{2}},$$

where $P_{\text{in}}$ is the input optical power, η is the optical transfer efficiency for the laser aperture to the focal plane, NA is the numerical aperture of the focusing optics, $\lambda_{0}$ is the free‐space wavelength of the laser (785 nm), and $n_{0}$ is the refractive index of the medium. Our calculations considered 90% optical power transfer efficiency (η=0.9) and water as the medium ($n_{0}=1.33$). Accordingly, throughout this work, “low‐power” refers to the incident laser power $P_{\text{in}}$; the local irradiance $I_{0}$ at the trapping plane can be substantially higher because the beam is concentrated by the focusing optics.

### 4.9 Figure‐of‐Merit Calculations

The figure of merit FoM is defined as:

(15) $$\text{FoM}=\log_{10}\left(S\cdot H\cdot R\cdot P\right),$$

where S, R, H, and P are stability, fixed‐wavelength force‐retention ratio, intensity‐based optical‐load and material‐response terms, respectively. We use this definition as a heuristic comparison metric against similar plasmonic nanotrapping reports [3, 10, 19, 30, 54, 61, 62, 68, 69, 70, 71, 72, 73, 74, 75, 76, 77, 78]. See Supporting Information S1: Table S1.

The multiplicative aggregation is adopted to represent the mutual contribution each parameter has for optimal performance; a low value of any individual factor reduces the overall FoM and an improvement on one factor alone does not necessarily improve the performance.

The trap‐stability factor S combines Brownian‐retention and local trap stiffness [1, 67]:

(16) $$S=B_{z}K_{xy},$$

where, $B_{z}$ describes axial Brownian‐retention and $K_{xy}$ describes lateral confinement. This definition follows the standard optical‐tweezer description in which trap depth relative to $k_{B}T$ describes resistance to thermally driven escape, whereas stiffness describes local confinement near equilibrium [1, 7, 11]. The axial Brownian‐retention factor B is defined as:

(17) $$B_{z}=\frac{\Delta U/\left(k_{B}T\right)}{\Delta U/\left(k_{B}T\right)+10},$$

where ΔU is the axial trap depth or estimated potential depth. This bounded definition gives $B_{z}=0.5$ when $\Delta U_{z}=10k_{B}T$ and approaches unity for deeper axial traps. A threshold of $10k_{B}T$ represents the practical Brownian‐retention criterion, consistent with the common use of $k_{B}T$‐scaled potential depths for evaluating optical trapping stability [11]. For the present work, ΔU was obtained directly from the full‐field force‐derived potential Equation (7). For literature entries where potential depth was not reported, $B_{z}=0.5$ was assigned an assumed reference value for the baseline comparison; these cases are explicitly identified as estimated benchmark entries. The lateral‐confinement factor was calculated as:

(18) $$K_{xy}=\frac{k_{xy}}{k_{xy}+k_{\text{ref}}},$$

where $k_{xy}$ is the lateral stiffness defined in Equation (10) and $k_{\text{ref}}$ is the reference stiffness set to $k_{\text{ref}}=1\;\text{fN}/\text{nm}.$ When both $k_{x}$ and $k_{y}$ were reported, $k_{xy}=\sqrt{k_{x}k_{y}}$ was used. When only one representative stiffness value was reported, that value was used as $k_{xy}$. When stiffness was not reported, $k_{xy}=k_{\text{ref}}$ was assigned, giving $K_{xy}=0.5$. These entries were marked as estimated benchmark cases in supporting information.

The intensity‐based optical‐load factor H is introduced as a bounded penalty for operating conditions that require larger incident optical irradiance [60, 79, 80]:

(19) $$H=\frac{I_{\text{ref}}}{I+I_{\text{ref}}},$$

where I is the incident optical irradiance and $I_{\text{ref}}=1.7$ mW/μm^2^ was selected as a practical reference intensity because temperature increases on the order of 10K have been measured in gold nanoaperture optical traps at comparable incident irradiances [60]. Importantly, H is not a calculation of local temperature rise, a thermal‐damage threshold, or a measure of biological safety. Local temperature rise depends on structure‐specific optical absorption, heat dissipation, and the surrounding thermal environment [60, 79, 80, 81]. The present factor therefore serves only as an intensity‐based comparative penalty within the heuristic FoM.

The fixed‐wavelength force‐retention ratio describes how much of the attractive axial force remains available at the fixed operating laser wavelength, $\lambda_{\text{laser}}=785$ nm [54]. In the ideal case, when force spectra were available, the same term R was calculated from the fixed‐wavelength force‐retention ratio:

(20) $$R=\frac{|F_{z}\left(\lambda_{0}\right)|}{|F_{z,\max}|},$$

where $F_{z}\left(\lambda_{\text{laser}}\right)$ is the axial force at the operating laser wavelength and $F_{z,\max}$ is the maximum attractive axial force obtained from the wavelength‐dependent force spectrum, typically near the particle‐shifted LSPR. For literature entries where wavelength‐dependent force‐retention data were not available, R=1 was assigned as a nonpenalizing placeholder for the baseline comparison, so that those studies were not additionally penalized for unavailable spectral force information. This parameter directly rewards structures that retain attractive axial force at the laser line.

The material‐response term P was calculated from the wavelength‐dependent nanoparticle complex polarizability and is defined as [26, 47, 82]:

(21) $$P=\frac{|\text{Re}\left[\hat{\alpha }\left(\lambda \right)\right]|}{1+|\text{Im}\left[\hat{\alpha }\left(\lambda \right)\right]|},$$

where $\hat{\alpha }$ is derived from Equation (4) as $\hat{\alpha }\left(\lambda \right)=\alpha \left(\lambda \right)/4\pi {\varepsilon_{0}\varepsilon_{m}a}^{3}$. The material‐response term P accounts for the fact that nanoparticles with different optical constants do not couple equally to the same near‐field distribution. The real part of polarizability is associated with the conservative gradient‐force response, whereas the imaginary part is associated with absorption and dissipative loss [50, 51]. This distinction is important when comparing dielectric (P=0.125) and gold (P=1.234) nanoparticles, because gold nanoparticles can provide strong optical coupling but also introduce significant absorption near their plasmonic response [33, 47, 64, 65, 83]. This parameter rewards materials with strong gradient‐force response while penalizing excessive absorptive loss. The term P is therefore not an empirical weight; it is a compact representation of the material‐dependent optical force budget. It also helps prevent direct comparison between dielectric and gold nanoparticles from being based only on intensity or field enhancement. Because the material‐response term P depends explicitly on the nanoparticle optical properties, the resulting score characterizes a platform‐particle operating pair rather than the trapping platform alone.

Because S, H, and R are bound to unity, whereas P is material and wavelength dependent with values larger than unity for metallic nanoparticles, the upper bound of the FoM is dictated primarily by $\log_{10}\left(P\right)$; therefore, the numerical FoM should be interpreted relative to this material‐dependent upper scale rather than to a universal zero or unity range.

### 4.10 Data Processing and Postanalysis

All exported electromagnetic fields, force components, detuning values, optical potentials, stiffness values, Brownian‐retention, power maps, and FoM datasets were processed in MATLAB R2023b (The MathWorks Inc.). The same postprocessing workflow was applied to all nanoparticle materials, nanoparticle sizes, wavelengths, and nanoparticle positions.

## Author Contributions

**Nasrin Abdollahi:** methodology, software, investigation, formal analysis, visualization, writing – original draft, writing – review and editing. **Abraham Vázquez‐Guardado:** conceptualization, formal analysis, supervision, writing – review and editing.

## Funding

This work was supported by the North Carolina State University start‐up funds Grant 201473‐02139.

## Conflicts of Interest

The authors declare no conflicts of interest.

## Supporting information


Supporting Information S1

## Data Availability

The data that support the findings of this study are available from the corresponding author upon reasonable request.
