# Supplementary material for: Engineering Low‐Power Cavity‐Coupled Plasmonic Nanotweezers
Source: Nanophotonics. 2026 Oct 11;15(19):e70318. doi: 10.1002/nap2.70318 (PMC13655230; doi:10.1002/nap2.70318)
Supplement: Supplementary file 1 [file NAP2-15-e70318-s001.docx]

**Supplementary Information**

**Engineering Low-Power Cavity-Coupled Plasmonic Nanotweezers**

*Nasrin Abdollahi^1^, and Abraham Vázquez-Guardado^1,2,^**

*^1^Department of Electrical and Computer Engineering, North Carolina State University, Raleigh, NC 27606, USA*

*^2^Center for Advanced Self-Powered Systems of Integrated Sensors and Technologies (ASSIST), North Carolina State University, Raleigh, NC 27695, USA*

***[*abraham.vg@ncsu.edu*](mailto:abraham.vg@ncsu.edu)

**Abstract:** Plasmonic nanoapertures extend optical trapping into the subwavelength regime but quantitative design rules remain limited because trapping is often inferred from spectral responses or field enhancement alone. This work establishes a full-field analysis framework for designing plasmonic nanoaperture tweezers under coherent illumination and experimental restrictions. We studied the plasmonic responses of three complementary plasmonic platforms –nanohole, nanohole-nanodisk, and cavity-coupled nanohole-nanodisk arrays– and their nanotrapping performance on dielectric and metallic nanoparticles using three-dimensional electromagnetic simulations and Maxwell stress tensor calculations. The results show that the force landscape in the particle volume depends strongly on the plasmonic resonance strength, the nanoparticle material, and the interaction with the target nanoparticle. In particular, we found that the cavity-coupled plasmonic array represents the most promising nanotrapping platform, capable of stable nanotrapping of metallic nanoparticles even under low power budgets and modest optical systems. Finally, we introduce a multi-criteria figure of merit (FoM) to compare trade-offs among trap stability, particle detuning effects, optical load, and nanoparticle material response. These results provide design guidance for compact, low-input-power plasmonic nanotweezer devices.

KEYWORDS: Optical trapping, Plasmonic nanotweezers, Maxwell stress tensor, Optical forces, Optical potential, Brownian retention.

Table of content

[1. Spectral and spatial near-field response of the CCnHnD.	3](#_Toc240540065)

[2. Benchmarking Dataset for Figure 4j	4](#_Toc240540066)

[Table S1. Literature benchmark inputs used for the FoM comparison in Figure 4j.	5](#_Toc240540067)

[Table S2. Calculated FoM components used for Figure 4j.	7](#_Toc240540068)

[References	8](#_Toc240540069)

# 1. Spectral and spatial near-field response of the CCnHnD.

To identify which cavity-coupled nanohole-nanodisk structure (CCnHnD) resonances generate experimentally useful near-fields for optical trapping, we examined the spatial electric-field distributions at the main absorption peaks. Figure S1 shows the absorption spectrum of the CCnHnD structure together with the corresponding normalized field-intensity maps, $\mid\mathbf{E/}\mathbf{E}_{\mathbf{in}}\mid^{2}$, in both $xy$ and $xz$ cross sections. Although multiple strong resonances are supported by the CCnHnD geometry, not all of them localize the field at the upper trapping interface. In particular, the resonances near $\sim670$ nm and $\sim900$ nm are mainly associated with cavity-coupled hybrid LSPR modes whose fields are buried within or below the cavity region. Therefore, these modes contribute strongly to absorption but are less relevant for nanotrapping because the enhanced fields are not accessible to nanoparticles above the structure.

**Figure** S1. Absorption spectrum of the CCnHnD showing three representative resonant modes labeled (1)-(3). The corresponding normalized electric-field intensity distributions, $|\mathbf{E/}\mathbf{E}_{\mathbf{in}}|^{2}$, are shown in $xy$ and $xz$ cross sections for each resonance. Dashed white outlines indicate the nanostructure and cavity boundaries, and red arrows indicate the incident electric-field polarization. The field maps show that the resonances near $\sim670$ nm and $\sim900$ nm are mainly localized within or below the cavity region, whereas the resonance near the target operating wavelength produces stronger field localization near the particle-accessible upper aperture region. Therefore, the buried cavity-coupled modes contribute to absorption but are less relevant for trapping nanoparticles positioned above the structure. Each field map is plotted with its own color scale to emphasize the spatial distribution of the corresponding mode.

# 2. Benchmarking Dataset for Figure 4j

The FoM equations and term definitions are provided in the Methods section of the main text. This Supporting Information provides the benchmark dataset and implementation notes used to generate Figure 4j, using the FoM definition. More favorable benchmarking cases appear closer to zero, whereas more negative values indicate reduced performance in one or more FoM components.

## Table S1. Literature benchmark inputs used for the FoM comparison in Figure 4j.

The table lists the study type, nanoparticle material, nanoparticle diameter, illumination wavelength, reported incident power or optical intensity, reported or inferred stiffness, and source reference. When values were not reported in the original study, they are marked as N/R. When a neutral or conservative placeholder was required for FoM calculation, the assumption is stated below the table.

**Table S1.** Literature benchmark inputs used for the FoM comparison in Figure 4j.

| **Ref.** | **Exp./Sim.** | **NP material** | **NP diameter (nm)** | **Illumination wavelength (nm)** | **Incident power (**$\mathbf{mW}$**)** | **Intensity (**$\boldsymbol{mW/\mu m}$**²)** | **Stiffness** $\boldsymbol{k}_{\boldsymbol{x}}$**/**$\boldsymbol{k}_{\boldsymbol{y}}$**(fN/nm)** |
| --- | --- | --- | --- | --- | --- | --- | --- |
| [1] | Sim. | Polystyrene | 4880 | 710 | 250 | 0.0025 | 0.027/0.022 |
| [2] | Exp. | Polystyrene | 200 | 974 | 5 | 2.5 | 19/4.8 |
| [3] | Exp. | Polystyrene | 200 | 1064 | 155 | 4000 | 13 |
| [4] | Exp. | Polystyrene | 100/500 | 980 | 3.5 | 2.8 | N/R |
| [5] | Sim. | Polystyrene | 50 | 1064 | 1.9 | 2 | 7200 |
| [6] | Exp. | Silica | 12 | 975 | 7.2 | 7.2 | N/R |
| [7] | Sim/Exp. | Polystyrene | 20 | 1064 | $6.6$ | N/R | 0.2625/0.0801 |
| [8] | Exp. | Polystyrene | 30/1000 | 980 | $0.6$ | $1$ | 0.85 /1.07$mW^{-1}$ |
| [9] | Sim. | Polystyrene / HIV-scale particle | 100 | 1064 | $0.6$ | N/R | 2.3/2.0 |
| [10] | Exp. | Polystyrene | 1200 | 800 | $0.05$ | $20$ | 14$mW^{-1}$ |
| [11] | Exp. | Gold | 60 | 1064 | $10$ | $1$ | 2.4 |
| [12] | Sim. | Quantum dot | 30 | 1064 | N/R | $15.6$ | 0.42/0.07$mW^{-1}$ |
| [13] | Exp. | Polystyrene | 30 | 980 | N/R | $0.51$ | 0.85$mW^{-1}$ |
| [14] | Sim/Exp. | Polystyrene | 300 | 980 | $5.5$ | $3.57$ | 0.34 / 0.69$mW^{-1}$ |
| [15] | Exp. | Polystyrene | 400 | 1000 | $24.5$ | $24.5$ | 0.34$mW^{-1}$ |
| [16] | Sim. | Polystyrene | 250/350 | 720/900 | N/R | $83$ | 727 |
| [17] | Exp. | Polystyrene | 20 | 930 | N/R | 0.23 | 1.99 |
| [18] | Exp. | Gold | 20 | 930 | N/R | 0.61 | 1.78 |
| ***** | **Sim.** | **Polystyrene** | **100** | **785** | **N/A** | $\boldsymbol{10}$ | **150/74** |
| ****** | **Sim.** | **Gold** | **100** | **785** | **N/A** | $\boldsymbol{1}$ | **72/67** |

**Abbreviations:** N/R = not reported; N/A = not applicable.

*This work (d-NP); **This work (Au-NP).

**Assumptions used for the benchmarking dataset.** When both lateral stiffness components were available, the effective lateral stiffness was calculated as $k_{xy}=\sqrt{k_{x}k_{y}}$. When only one representative stiffness value was reported that value was used as $k_{xy}$. When stiffness was not reported, $k_{xy}=k_{\mathrm{ref}}=1 fN/nm$ was assigned, giving $K_{xy}=0.5$. When an axial trap depth or potential barrier was reported, it was used to calculate $B_{z}$. For literature entries where a compatible axial energy barrier was unavailable, $B_{z}=0.5$ was assigned as an assumed reference value for the baseline comparison; this value does not represent a measured property of the corresponding trapping platform. For literature entries where wavelength-dependent force-retention data were unavailable, $R=1$ was assigned as a non-penalizing placeholder. This assignment does not imply experimentally demonstrated perfect force retention; rather, it prevents the corresponding study from being penalized for spectral-force information that was not reported. Benchmark entries containing one or more assumed or placeholder quantities are therefore identified as estimated cases and should not be interpreted as fully reported experimental or simulation FoM values. The present-work entries use the directly calculated values reported in the main manuscript.

## Table S2. Calculated FoM components used for Figure 4j.

The table reports the optical-load factor term $H$, trap-stability factor $S$, fixed-wavelength force-retention ratio $R$, material-response term $P$, the raw product $SHRP$, and the corresponding figure of merit, $\mathrm{FoM}=\log_{10}(SHRP)$.

**Table S2.** Calculated FoM components used for Figure 4j.

| **Ref.** | $\boldsymbol{H}$ | $\boldsymbol{S}$ | $\boldsymbol{R}$ | $\boldsymbol{P}$ | $\boldsymbol{SHRP}$ | $\boldsymbol{Fo}\boldsymbol{M}\boldsymbol{}$ |
| --- | --- | --- | --- | --- | --- | --- |
| [1] | 9.985E-01 | 1.896E-02 | 1 | 1.252E-01 | 2.370E-03 | -2.63 |
| [2] | 4.048E-01 | 4.526E-01 | 1 | 1.252E-01 | 2.294E-02 | -1.64 |
| [3] | 4.248E-04 | 4.642E-01 | 1 | 1.252E-01 | 2.469E-05 | -4.61 |
| [4] | 3.778E-01 | 2.500E-01 | 1 | 1.252E-01 | 1.182E-02 | -1.93 |
| [5] | 4.595E-01 | 5.000E-01 | 1 | 1.252E-01 | 2.876E-02 | -1.54 |
| [6] | 1.910E-01 | 2.500E-01 | 1 | 8.312E-02 | 3.969E-03 | -2.40 |
| [7] | 2.048E-01 | 6.332E-02 | 1 | 1.252E-01 | 1.624E-03 | -2.79 |
| [8] | 6.296E-01 | 2.440E-01 | 1 | 1.252E-01 | 1.923E-02 | -1.72 |
| [9] | 7.391E-01 | 3.410E-01 | 1 | 1.252E-01 | 3.156E-02 | -1.50 |
| [10] | 7.834E-02 | 4.666E-01 | 1 | 1.252E-01 | 4.577E-03 | -2.34 |
| [11] | 6.296E-01 | 3.529E-01 | 1 | 1.116E+00 | 2.480E-01 | -0.61 |
| [12] | 9.827E-02 | 7.166E-02 | 1 | 3.877E-01 | 2.730E-03 | -2.56 |
| [13] | 7.692E-01 | 2.297E-01 | 1 | 1.252E-01 | 2.212E-02 | -1.66 |
| [14] | 3.226E-01 | 1.631E-01 | 1 | 1.252E-01 | 6.587E-03 | -2.18 |
| [15] | 6.489E-02 | 1.268E-01 | 1 | 1.252E-01 | 1.030E-03 | -2.99 |
| [16] | 2.007E-02 | 4.999E-01 | 1 | 1.252E-01 | 1.256E-03 | -2.90 |
| [17] | 8.808E-01 | 3.328E-01 | 1 | 1.252E-01 | 3.670E-02 | -1.44 |
| [18] | 7.359E-01 | 3.202E-01 | 1 | 1.152 E+00 | 2.315E-01 | -0.69 |
| This work (d-NP) | 1.453E-01 | 4.954E-01 | 0.85 | 1.252E-01 | 7.660E-03 | -2.12 |
| This work (Au-NP) | 6.296E-01 | 4.999E-01 | 0.6 | 1.234E+00 | 2.330E-01 | -0.63 |

# References

[1]	Righini M, Volpe G, Girard C, Petrov D, Quidant R. Surface Plasmon Optical Tweezers: Tunable Optical Manipulation in the Femtonewton Range. Phys Rev Lett 2008;100:186804. https://doi.org/10.1103/PhysRevLett.100.186804.

[2]	Wang K, Schonbrun E, Steinvurzel P, Crozier KB. Trapping and rotating nanoparticles using a plasmonic nano-tweezer with an integrated heat sink. Nat Commun 2011;2. https://doi.org/10.1038/ncomms1480.

[3]	Grigorenko AN, Roberts NW, Dickinson MR, Zhang Y. Nanometric optical tweezers based on nanostructured substrates. Nat Photonics 2008;2:365–70. https://doi.org/10.1038/nphoton.2008.78.

[4]	Chen KY, Lee AT, Hung CC, Huang JS, Yang YT. Transport and trapping in two-dimensional nanoscale plasmonic optical lattice. Nano Lett 2013;13:4118–22. https://doi.org/10.1021/nl4016254.

[5]	Juan ML, Gordon R, Pang Y, Eftekhari F, Quidant R. Self-induced back-action optical trapping of dielectric nanoparticles. Nat Phys 2009;5:915–9. https://doi.org/10.1038/nphys1422.

[6]	Pang Y, Gordon R. Optical trapping of 12 nm dielectric spheres using double-nanoholes in a gold film. Nano Lett 2011;11:3763–7. https://doi.org/10.1021/nl201807z.

[7]	Xu Z, Song W, Crozier KB. Direct Particle Tracking Observation and Brownian Dynamics Simulations of a Single Nanoparticle Optically Trapped by a Plasmonic Nanoaperture. ACS Photonics 2018;5:2850–9. https://doi.org/10.1021/acsphotonics.8b00176.

[8]	Han X, Truong VG, Chormaic SN. Efficient microparticle trapping with plasmonic annular apertures arrays. Nano Futures 2018;2. https://doi.org/10.1088/2399-1984/aad4c0.

[9]	Pang Y, Song H, Kim JH, Hou X, Cheng W. Optical trapping of individual human immunodeficiency viruses in culture fluid reveals heterogeneity with single-molecule resolution. Nat Nanotechnol 2014;9:624–30. https://doi.org/10.1038/nnano.2014.140.

[10]	Roxworthy BJ, Toussaint KC. Femtosecond-pulsed plasmonic nanotweezers. Sci Rep 2012;2. https://doi.org/10.1038/srep00660.

[11]	Mestres P, Berthelot J, Acimovic SS, Quidant R. Unraveling the optomechanical nature of plasmonic trapping. Light Sci Appl 2016;5. https://doi.org/10.1038/lsa.2016.92.

[12]	El Eter A, Hameed NM, Baida FI, Salut R, Filiatre C, Nedeljkovic D, et al. Fiber-integrated optical nano-tweezer based on a bowtie-aperture nano-antenna at the apex of a SNOM tip. Opt Express 2014;22:10072. https://doi.org/10.1364/oe.22.010072.

[13]	Han X, Truong VG, Thomas PS, Chormaic SN. Sequential trapping of single nanoparticles using a gold plasmonic nanohole array. Photonics Res 2018;6:981. https://doi.org/10.1364/prj.6.000981.

[14]	Kang JH, Kim K, Ee HS, Lee YH, Yoon TY, Seo MK, et al. Low-power nano-optical vortex trapping via plasmonic diabolo nanoantennas. Nat Commun 2011;2. https://doi.org/10.1038/ncomms1592.

[15]	Kotsifaki DG, Kandyla M, Lagoudakis PG. Plasmon enhanced optical tweezers with gold-coated black silicon. Sci Rep 2016;6. https://doi.org/10.1038/srep26275.

[16]	Aqhili A, Darbari S. A numerical study on the closed packed array of gold discs as an efficient dual mode plasmonic tweezers. Sci Rep 2021;11. https://doi.org/10.1038/s41598-021-99633-x.

[17]	Kotsifaki DG, Truong VG, Chormaic SN. Fano-Resonant, Asymmetric, Metamaterial-Assisted Tweezers for Single Nanoparticle Trapping. Nano Lett 2020;20:3388–95. https://doi.org/10.1021/acs.nanolett.0c00300.

[18]	Bouloumis TD, Kotsifaki DG, Nic Chormaic S. Enabling Self-Induced Back-Action Trapping of Gold Nanoparticles in Metamaterial Plasmonic Tweezers. Nano Lett 2023;23:4723–31. https://doi.org/10.1021/acs.nanolett.2c04492.
